# Empiric use of anticoagulation in hospitalized patients with COVID-19: a propensity score-matched study of risks and benefits

**DOI:** 10.1186/s40364-021-00283-y

**Published:** 2021-05-01

**Authors:** Bo Yu, Victor Perez Gutierrez, Alex Carlos, Gregory Hoge, Anjana Pillai, J. Daniel Kelly, Vidya Menon

**Affiliations:** 1 Department of Medicine, New York City Health + Hospitals, Lincoln Medical Center, Bronx, New York USA; 2 Department of Epidemiology and Biostatistics, University of California San Francisco, San Francisco, California USA; 3 Department of Medicine, University of California San Francisco, San Francisco, California USA; 4 Institute of Global Health Science, University of California San Francisco, San Francisco, California USA; 5 F.I. Proctor Foundation, University of California San Francisco, San Francisco, California USA

## Abstract

**Background:**

Hospitalized patients with COVID-19 demonstrate a higher risk of developing thromboembolism. Anticoagulation (AC) has been proposed for high-risk patients, even without confirmed thromboembolism. However, benefits and risks of AC are not well assessed due to insufficient clinical data. We performed a retrospective analysis of outcomes from AC in a large population of COVID-19 patients.

**Methods:**

We retrospectively reviewed 1189 patients hospitalized for COVID-19 between March 5 and May 15, 2020, with primary outcomes of mortality, invasive mechanical ventilation, and major bleeding. Patients who received therapeutic AC for known indications were excluded. Propensity score matching of baseline characteristics and admission parameters was performed to minimize bias between cohorts.

**Results:**

The analysis cohort included 973 patients. Forty-four patients who received therapeutic AC for confirmed thromboembolic events and atrial fibrillation were excluded. After propensity score matching, 133 patients received empiric therapeutic AC while 215 received low dose prophylactic AC. Overall, there was no difference in the rate of invasive mechanical ventilation (73.7% versus 65.6%, *p* = 0.133) or mortality (60.2% versus 60.9%, *p* = 0.885). However, among patients requiring invasive mechanical ventilation, empiric therapeutic AC was an independent predictor of lower mortality (hazard ratio [HR] 0.476, 95% confidence interval [CI] 0.345–0.657, *p* < 0.001) with longer median survival (14 days vs 8 days, *p* < 0.001), but these associations were not observed in the overall cohort (*p* = 0.063). Additionally, no significant difference in mortality was found between patients receiving empiric therapeutic AC versus prophylactic AC in various subgroups with different D-dimer level cutoffs. Patients who received therapeutic AC showed a higher incidence of major bleeding (13.8% vs 3.9%, *p* < 0.001). Furthermore, patients with a HAS-BLED score of ≥2 had a higher risk of mortality (HR 1.482, 95% CI 1.110–1.980, *p* = 0.008), while those with a score of ≥3 had a higher risk of major bleeding (Odds ratio: 1.883, CI: 1.114–3.729, *p* = 0.016).

**Conclusion:**

Empiric use of therapeutic AC conferred survival benefit to patients requiring invasive mechanical ventilation, but did not show benefit in non-critically ill patients hospitalized for COVID-19. Careful bleeding risk estimation should be pursued before considering escalation of AC intensity.

**Supplementary Information:**

The online version contains supplementary material available at 10.1186/s40364-021-00283-y.

## Introduction

The global pandemic of SARS-CoV-2 infection with resulting coronavirus disease 2019 (COVID-19) has resulted in worldwide devastating health crises [1, 2]. Hemostatic abnormalities have been seen in patients with COVID-19, including elevated D-dimer levels, thrombocytopenia, increased fibrin degradation products, disseminated intravascular coagulation (DIC), and thromboembolism [1, 3–9]. The reported incidence of thromboembolic events can be 20–30% in various retrospective studies [10, 11]. Furthermore, these abnormalities were associated with higher rates of mechanical ventilation, intensive care unit (ICU) admission, and death [5].

During the early phase of the COVID-19 pandemic, and with evolving knowledge about related coagulopathies and thrombosis complications, there was a lack of consensus about appropriate anticoagulation (AC) intensity. Initially, prophylactic intensity of AC was recommended for all patients hospitalized for COVID-19 [12]. However, increasing evidence demonstrated that patients developed venous thromboembolism (VTE) despite the standard pharmacological prophylaxis, especially in critically ill patients [11, 13]. Therefore, empiric use of higher intensity therapeutic AC was proposed for patients with elevated D-dimer values, even without confirmed thromboembolism [11, 14–16]. However, safety concerns arose with evidence of increased bleeding risk in certain patients, thus there appears to be an unclear benefit with empiric therapeutic AC in COVID-19 patients with high pro-thrombotic risks [17, 18].

As New York City (NYC) became the epicenter of the COVID-19 pandemic in March 2020, our hospital in the South Bronx admitted and managed SARS-CoV-2 infections ranging from moderate to severe and critical disease. Therapeutic AC was used empirically at the discretion of the treating physicians when there was a high clinical suspicion of thrombosis or embolism with elevated D-dimers. We aimed at assessing the impact of empiric use of therapeutic AC on the outcome of a large cohort of hospitalized patients with COVID-19.

## Methods

### Study design and patient population

This is a retrospective cohort study conducted in a tertiary, acute care hospital in South Bronx, New York. We included all hospitalized adults with a positive SARS-CoV-2 PCR result from March 5 to May 15, 2020. Patients who were excluded from the study were those who died or were discharged within 48 h after admission, those who were transferred to another facility, those who were on long-term AC before the admission, and those who received thrombolytics during the hospital course. Follow-up continued through July 1, 2020, when all participants had confirmed outcomes (discharged alive versus death). This work was approved by our hospital Institutional Review Board (IRB: 20–017).

Therapeutic anticoagulants included enoxaparin (1 mg/kg twice daily), apixaban (≥ 5 mg twice daily), unfractionated heparin (UFH) infusion, and fondaparinux (≥ 5 mg once daily). The indications of empiric therapeutic AC were persistently high oxygen requirement with elevated D-dimer more than 6 times the upper limit of normal or age-adjusted D-dimer cutoff (age in years x 10mcg/L for patients over 50 years old) [19]. Those who received therapeutic AC for known indications, including confirmed VTE (pulmonary embolism [PE], deep venous thrombosis [DVT]), arterial thrombotic events (acute coronary syndrome, acute ischemic stroke, and acute peripheral artery thrombosis), and atrial fibrillation, were excluded from the final analysis.

### Data collection and definition of variables

Sociodemographic and broad characteristics including age, sex, ethnicity, and comorbidities (hypertension, diabetes, chronic lung disease, chronic kidney disease, congestive heart disease, chronic liver disease, cancer, HIV, transplant, and other immunosuppression conditions) and presenting symptoms were collected from medical records. The Charlson Comorbidity Index (CCI), was used as a measure of aggregate comorbidity burden [20]. Sepsis was determined by a quick SOFA score (altered mental status, respiratory rate ≥ 22, and/or systolic blood pressure ≤ 100) ≥2 on admission [21]. The severity of COVID-19 infection on admission was classified into three categories (moderate, severe, critical) according to World Health Organization (WHO) guidelines. Moderate cases were defined as hospitalized patients with any of the following: fever, cough, or shortness of breath with radiographic evidence of pulmonary infiltrates and O2 saturation > 94% on room air. Severe cases were defined as hospitalized patients with respiratory rate (RR) ≥24/min, O2 saturation ≤ 94%, PaO2/FiO2 < 300, and/or lung infiltrates involving > 50% of lung fields within 24–48 h. Critical cases were defined as respiratory failure requiring mechanical ventilation, shock, or organ failure [22]. Bleeding risk was evaluated by HAS-BLED score including Hypertension, Abnormal renal/liver function, Stroke, Bleeding, Labile International Normalized Ratio (INR), Elderly, Drugs or alcohol use [23]. Major bleeding was defined as a fall in hemoglobin of 2 g/dL or more, requirement of 2 or more units of red blood cell transfusion, or any observed bleeding including intracranial hemorrhage, melena, hematemesis, hemoptysis, hematuria, etc. [24].

### Statistical analysis

Intergroup comparison of categorical variables was performed by Pearson’s Chi-square or the Fisher Exact test. Mann-Whitney U test was used to compare nonparametric continuous variables. To reduce potential confounders, we performed propensity score matching (PSM) using a 1:2 ratio with the nearest neighbor matching procedure and a caliber of 0.3 without replacement [25]. Baseline characteristics, initial symptoms, laboratory and radiographic findings, other medications, as well as the assessment of COVID-19 severity, sepsis, and acute kidney injury, were adjusted by PSM (Table 1). Adequate matching for the major imbalance of each covariate was fully assessed by visually comparing the distribution of propensity score and standardized difference (Figure S1). Univariate and multivariate cox proportional regression models were adopted to explore predictors of survival expressed as hazard ratio (HR) and 95% confidence interval (CI). To minimize immortal bias, empiric therapeutic AC was defined as a time-dependent covariate, adjusted by time to the start of exposure. Kaplan-Meier curves were plotted for matched patients with therapeutic AC versus prophylactic AC. Logistic regression analysis was used to determine the association between AC intensity and mortality stratified by D-dimer, as well as between HAS-BLED score and risk of major bleeding. *P* < 0.05 was considered statistically significant. All data analysis was conducted using SPSS for Windows, version 22.0 (IBM), and R software, version 3.6.1 (R Project for Statistical Computing).

**Table 1:** Characteristics of all hospitalized COVID-19 patients receiving empiric therapeutic AC versus prophylactic AC

	Prior to Propensity Score Match			After Propensity Score Match		
Parameters	Therapeutic AC (***n*** = 165)	Prophylactic AC (***n*** = 764)	***p*** value	Therapeutic AC (***n*** = 133)	Prophylactic AC (***n*** = 215)	***p*** value
Age, median (IQR)	61 (54–72)	62 (50–75)	0.894	62 (54–72)	65 (52–75)	0.345
Age categories, n (%)			0.001*			0.064
18–39	11 (5.3)	87 (11.4)		6 (4.5)	19 (8.8)	
40–59	78 (37.3)	244 (31.9)		50 (37.6)	61 (28.4)	
60–79	101 (48.3)	309 (40.4)		65 (48.9)	101 (47.0)	
≥80	19 (9.1)	124 (16.2)		12 (9.0)	34 (15.8)	
Gender, Female, n (%)	77 (36.8)	336 (44.0)	0.064	47 (35.3)	80 (37.2)	0.725
Ethnicity, n (%)			0.348			0.737
Latinx	141 (67.5)	482 (63.0)		97 (72.9)	154 (71.6)	
Black	62 (29.7)	236 (31.0)		34 (25.6)	55 (25.6)	
White	4 (1.9)	29 (3.8)		2 (1.5)	6 (2.8)	
Asian	2 (1.0)	17 (2.2)		0	0	
Body Mass Index, median (IQR)	30.1 (26.2–35.0)	29.0 (25.0–33.7)	0.066	30.2 (26.4–35.0)	29.4 (25.0–34.2)	0.155
Body Mass Index categories, n (%)			0.136			0.258
Underweight	1 (0.6)	14 (1.8)		1 (0.8)	5 (2.3)	
Normal	28 (17.0)	170 (22.3)		21 (15.8)	42 (19.1)	
Overweight	49 (29.7)	244 (31.9)		39 (29.3)	74 (34.4)	
Obesity	107 (51.2)	327 (42.8)		72 (54.1)	95 (44.2)	
Current smoker, n (%)	4 (1.9)	36 (4.7)	0.071	1 (0.8)	3 (1.4)	1.000
Past diagnosis, n (%)						
Diabetes mellitus	94 (45.0)	358 (46.9)	0.629	55 (41.4)	91 (42.3)	0.858
Hypertension	120 (57.4)	322 (42.1)	< 0.001*	67 (50.4)	102 (47.4)	0.595
Chronic lung disease	34 (16.3)	148 (19.4)	0.308	24 (18.0)	39 (18.1)	0.982
Congestive heart failure	8 (3.8)	55 (7.2)	0.049*	6 (4.5)	12 (5.6)	0.661
Chronic kidney disease, stage> 3	24 (11.5)	105 (13.7)	0.393	12 (9.0)	23 (10.7)	0.614
Cancer	19 (9.1)	52 (6.8)	0.260	10 (7.5)	19 (8.8)	0.665
Chronic liver disease and Cirrhosis	9 (5.5)	20 (2.6)	0.047*	6 (4.5)	10 (4.7)	0.952
HIV, transplant, and other immunosuppression status	7 (4.2)	34 (4.5)	0.906	7 (5.3)	11 (5.1)	0.952
Charlson Comorbidities Index (CCI), median (IQR)	3 (1–4)	3 (1–5)	0.909	2 (1–4)	3 (1–4)	0.976
HAS-BLED score, median (IQR)	2 (1–3)	2 (1–4)	0.061	2 (1–2)	2 (1–3)	0.109
Initial symptoms, n (%)						
Fever	127 (60.8)	461 (60.3)	0.911	83 (62.4)	129 (60.0)	0.655
Cough	148 (70.8)	497 (65.1)	0.118	97 (72.9)	149 (69.3)	0.470
Dyspnea	166 (79.4)	520 (68.1)	0.001*	107 (80.5)	167 (77.7)	0.538
Gastrointestinal symptoms	33 (15.8)	205 (26.8)	0.001*	24 (18.0)	51 (23.7)	0.211
Neurologic symptoms	37 (17.7)	132 (17.3)	0.885	22 (16.5)	45 (20.9)	0.313
Initial laboratory test, median (IQR)						
White blood cell count [4.8–10.8 × 10 ^3^/μL]	10.0 (7.0–13.2)	7.6 (5.5–10.4)	< 0.001*	9.8 (6.9–12.5)	9.0 (6.6–11.5)	0.165
Absolute lymphocyte cell count [1.0–4.8 × 10 ^3^/μL]	1.0 (0.6–1.3)	1.0 (0.7–1.4)	0.027*	0.9 (0.6–1.3)	0.9 (0.7–1.3)	0.428
Absolute neutrophil cell count [1.8–7.8 × 10 ^3^/μL]	8.5 (5.6–11.3)	5.7 (3.9–8.4)	< 0.001*	8.4 (5.3–10.6)	7.2 (4.9–9.5)	0.077
Hemoglobin [14–18 g/dL]	13.2 (11.9–14.3)	13.2 (11.6–14.5)	0.864	13.0 (11.6–14.1)	13.1 (11.5–14.6)	0.467
Platelet count [150–450/μL]	248.0 (194.0–312.0)	215.0 (167.0–275.0)	< 0.001*	241.0 (192.5–307.5)	232.0 (177.0–310.0)	0.462
D-dimer [<=230 ng/mL]	1207.0 (413.0–5721.0)	498.0 (258.0–897.0)	< 0.001*	882.0 (403.0–3278.5)	793.0 (393.0–2024.1)	0.051
Lactate dehydrogenase [135–225 U/L]	592.5 (457.0–768.8)	457.0 (333.3–640.8)	< 0.001*	599.0 (459.0–724.5)	605.0 (398.0–715.0)	0.597
C-reactive protein [0–0.40 mg/dL]	19.1 (10.8–30.0)	11.3 (5.5–21.3)	< 0.001*	20.7 (11.2–27.1)	18.3 (10.8–26.5)	0.368
Interleukin-6 [0–5.5 pg/mL]	129.5 (62.1–285.8)	78.3 (27.1–172.3)	< 0.001*	221.7 (92.6–299.5)	238.6 (96.7–278.7)	0.249
Ferritin [12–300 ng/mL]	883.0 (492.0–1606.0)	688.0 (365.0–1224.5)	0.002*	1070.0 (502.5–1532.5)	1067.0 (562.0–1387.5)	0.568
Radiographic findings, n (%)						
Bilateral patchy infiltrates	131 (62.7)	412 (53.9)	0.024*	91 (68.4)	129 (60.0)	0.137
Ground glass opacity	82 (39.2)	217 (28.4)	0.003*	53 (39.8)	70 (32.6)	0.238
Consolidation	19 (9.1)	102 (13.4)	0.098	12 (9.0)	26 (12.1)	0.207
Atelectasis	4 (1.9)	33 (4.3)	0.107	2 (1.5)	7 (3.3)	0.491
Other medications received, n (%)						
Hydroxychloroquine	167 (79.9)	542 (70.9)	0.010*	105 (78.9)	165 (76.6)	0.589
Azithromycin	177 (84.7)	553 (72.4)	< 0.001*	111 (83.4)	173 (80.6)	0.480
Remdesevir	15 (7.2)	1 (0.1)	< 0.001*	1 (0.8)	1 (0.5)	0.555
Sarilumab/Tocilizumab	26 (12.4)	35 (4.6)	< 0.001*	15 (11.3)	20 (9.4)	0.540
Convalescent plasma	22 (10.5)	6 (0.8)	< 0.001*	7 (5.3)	6 (3.0)	0.122
Stress dose of steroid	55 (33.3)	74 (9.7)	< 0.001*	28 (18.6)	40 (18.4)	0.756
COVID-19 severity classification on admission, n (%)			< 0.001*			0.458
Moderate	21 (10.0)	378 (49.3)		13 (9.8)	32 (14.9)	
Severe	83 (39.7)	209 (27.4)		47 (35.3)	80 (37.2)	
Critical	105 (50.2)	177 (23.2)		73 (54.9)	103 (47.9)	
Sepsis by quick SOFA on admission, n (%)	37 (22.4)	230 (30.1)	0.048*	35 (26.3)	51 (23.7)	0.586
Acute kidney injury on admission, n (%)	53 (32.1)	218 (28.5)	0.358	45 (33.8)	77 (35.8)	0.707
**Hospital course (unmatched)**						
Invasive mechanical ventilation, n (%)	128 (77.6)	221 (28.9)	< 0.001*	98 (73.7)	141 (65.6)	0.113
Major bleeding, n (%)	23 (14.1)	27 (3.5)	< 0.001*	18 (13.8)	8 (3.9)	< 0.001*
Days of hospitalization, median (IQR)	11 (6–21)	7 (4–12)	< 0.001*	9 (6–19)	7 (5–12)	< 0.001*
All-cause mortality, n (%)	83 (50.3)	165 (21.6)	< 0.001*	80 (60.2)	131 (60.9)	0.885

*Significant at *p* < 0.05

## Results

### Baseline characteristics and PSM

**Fig. 1:**
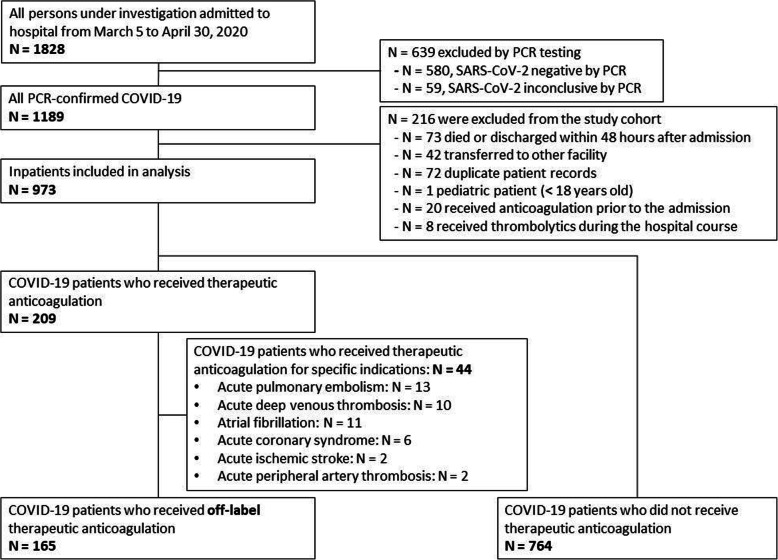
Flow diagram of process for patient inclusion as participants in the study

Of the 1189 consecutive patients hospitalized with confirmed COVID-19 infection, 973 patients were included in the study. 209 (21.5%) patients received therapeutic AC and 764 (78.5%) received prophylactic AC. In the therapeutic AC group, 165 individuals empirically received full dose AC, of which 44 received higher intensity AC for specific indications (such as PE, etc., as mentioned above) and were excluded from our final analysis. A total of 28 (2.9%) thromboembolic events were confirmed including 13 incidents of acute PE, 11 occurrences of DVT, and two individuals suffered from acute ischemic strokes, and 2 experienced acute peripheral artery occlusions (Fig. 1). The median duration of AC treatment was 5 days (IQR: 3–8 days). And the median time from hospital admission to the start of AC was 3 days (IQR: 0–5 days). 76.3% of patients of those treated with therapeutic AC received the first dose within 72 h after presentation to the emergency room.

Table 1 demonstrated baseline cohort characteristics. Prior to PSM, empiric AC exposure differed between age groups, comorbidities, initial symptoms, laboratory, and radiographic findings. Patients in the therapeutic AC group had a higher prevalence of severe and critical COVID-19 and higher levels of inflammatory markers on admission. After PSM, 133 patients were included in the empiric therapeutic AC cohort while 215 patients were in the prophylactic AC cohort. All covariates were balanced between the two groups after PSM, supported by closed standard mean differences (Figure S2).

### Primary outcome and risk factor analysis

Prior to PSM stratification, 83 of 165 (50.3%) patients treated with therapeutic AC died, with a median hospital stay of 11 days compared to 165 of 764 (21.6%) deaths and a median hospital stay of 7 days in patients receiving prophylactic AC. After PSM stratification, no statistical difference between the two groups was found when assessing for rates of invasive mechanical ventilation (73.7% versus 65.6%, *p* = 0.133) and mortality (60.2% versus 60.9%, *p* = 0.885). Also, the median duration of hospitalization was prolonged in patients treated with therapeutic AC (9 days versus 7 days, *p* < 0.001). Yet, patients receiving therapeutic AC showed a much higher risk of major bleeding (13.8% vs 3.9%, *p* < 0.001) (Table 1).

**Fig. 2:**
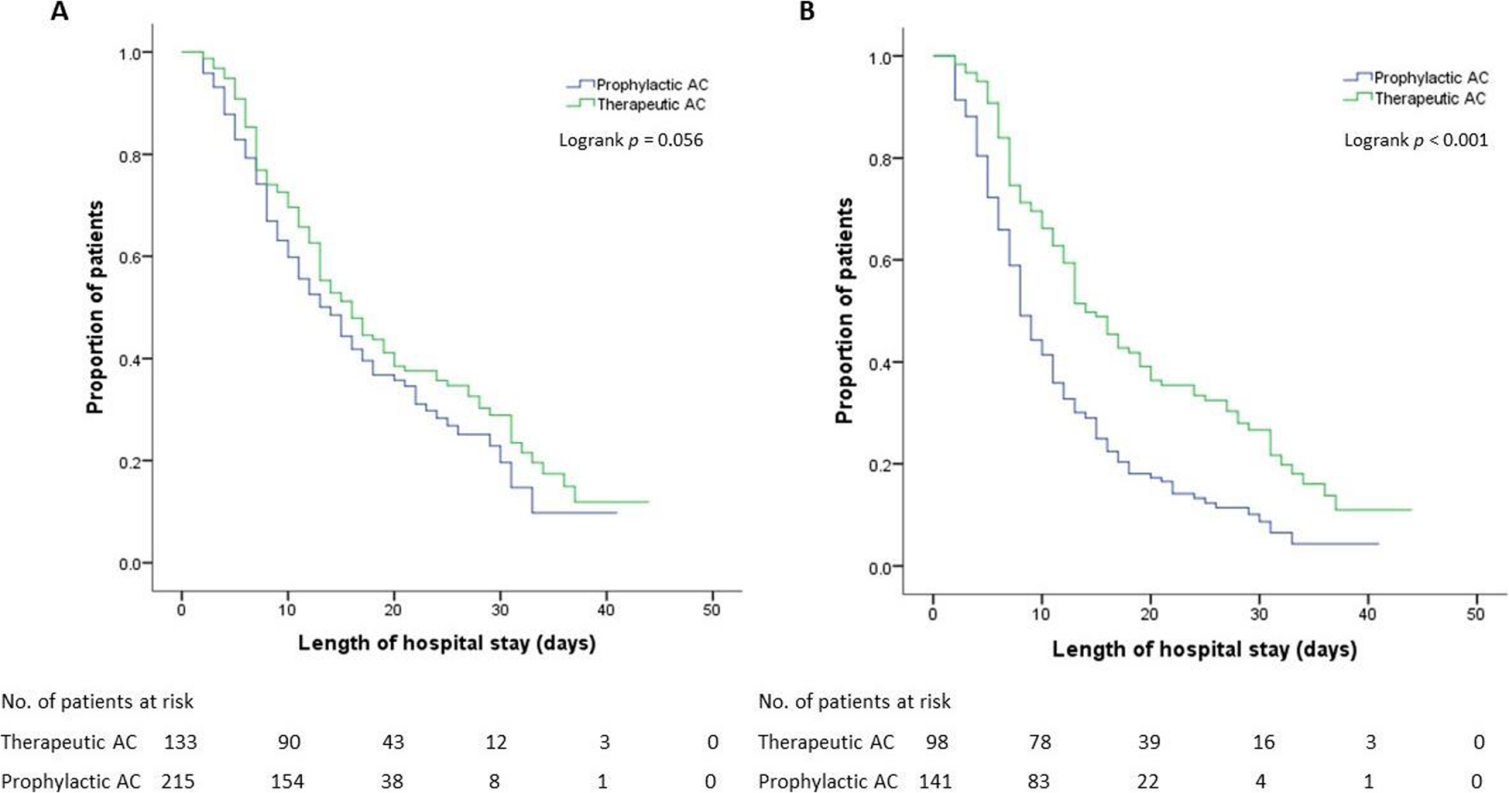
Kaplan Meier Curve for all hospitalized patients (**a**) and patients requiring invasive mechanical ventilation (**b**) with COVID-19 according to receiving therapeutic AC versus prophylactic AC

Among patients requiring invasive mechanical ventilation, those receiving empiric therapeutic AC showed lower mortality compared to those receiving prophylactic AC only (75.5% vs 83.7%, *p* < 0.001). After adjusting for baseline characteristics on admission, therapeutic AC remained an independent predictor of improved survival (adjusted hazard ratio [aHR] 0.476, 95% confidence interval [CI] 0.345–0.657, *p* < 0.001) among intubated patients. However, empiric therapeutic AC had no significant correlation to mortality (*p* = 0.063) when evaluating the entire cohort. Besides, advanced age, critical COVID-19 infection, and sepsis on admission appeared to be independent predictors of all-cause mortality regardless of invasive mechanical ventilation (Table 2). Figure 2a displayed the Kaplan-Meier survival curve for all the patients. There was no significant difference in the median survival between the two AC cohorts (*p* = 0.056). As indicated in Fig. 2b with a Kaplan-Meier survival curve for intubated patients only, those receiving therapeutic AC had a significantly longer median survival compared to those without therapeutic AC (14 days vs 8 days, *p* < 0.001).

**Table 2:** Cox proportional hazards model of risk factors for all-cause mortality among patients with or without receiving empiric therapeutic AC

	All matched patients					Patients requiring invasive mechanical ventilation				
	Death/N at risk (%)	Univariate analysis		Multivariate analysis		Death/N at risk (%)	Univariate analysis		Multivariate analysis	
		HR (95% CI)	***p*** value	HR (95% CI)	***p*** value		HR (95% CI)	***p*** value	HR (95% CI)	***p*** value
**Empiric therapeutic AC**										
**No**	131/215 (60.9)	Reference				118/141 (83.7)	Reference		Reference	
**Yes**	80/133 (60.2)	0.792 (0.619–1.013)	0.063			74/98 (75.5)	0.536 (0.413–0.695)	< 0.001*	0.476 (0.345–0.657)	< 0.001*
**Age**										
**< 65 years old**	103/199 (51.8)	Reference		Reference		98/138 (71.0)	Reference		Reference	
**≥ 65 years old**	108/149 (72.5)	1.423 (1.190–1.701)	< 0.001*	1.028 (1.014–1.142)	< 0.001*	90/101 (89.1)	1.276 (1.042–1.563)	0.008*	1.207 (1.006–1.526)	0.015*
**Gender**										
**Male**	133/221 (60.2)	Reference				119/151 (78.8)	Reference			
**Female**	78/127 (61.4)	0.915 (0.692–1.211)	0.536			73/88 (83.0)	0.920 (0.687–1.232)	0.577		
**Ethnicity**										
**Non-Latinx**	56/97 (57.7)	Reference				51/64 (79.7)	Reference			
**Latinx**	155/251 (61.8)	0.930 (0.709–1.221)	0.603			141/175 (80.6)	1.082 (0.806–1.453)	0.598		
**Body-mass index**										
**< 30**	104/181 (57.4)	Reference				87/111 (78.4)	Reference			
**≥ 30**	107/167 (64.1)	1.011 (0.995–1.027)	0.188			105/128 (82.0)	1.002 (0.986–1.018)	0.818		
**Comorbidities**										
**Diabetes mellitus**	91/146 (62.3)	1.087 (0.828–1.428)	0.548			81/102 (79.4)	0.996 (0.748–1.326)	0.975		
**Hypertension**	105/168 (62.1)	1.126 (0.860–1.475)	0.389			89/106 (84.0)	1.214 (0.914–1.613)	0.180		
**COPD/asthma**	45/63 (71.4)	1.429 (1.028–1.988)	0.034*			45/50 (90.0)	1.502 (1.074–2.101)	0.017*		
**Congestive heart failure**	11/18 (61.1)	1.196 (0.652–2.195)	0.564			10/11 (90.9)	1.196 (1.067–3.846)	0.031*		
**Chronic kidney disease**	23/35 (65.7)	1.196 (0.775–1.843)	0.419			17/22 (77.3)	0.899 (0.547–1.480)	0.676		
**Cancer**	21/29 (72.4)	1.341 (0.854–2.106)	0.202			16/19 (84.2)	1.000 (0.599–1.668)	1.000		
**CCI**										
**< 2**	66/120 (55.0)	Reference				65/84 (77.4)	Reference			
**≥ 2**	145/228 (63.6)	1.469 (1.000–2.799)	0.050			127/155 (81.9)	1.411 (0.908–2.931)	0.091		
**COVID-19 severity**										
**Moderate**	8/45 (17.8)	Reference		Reference		4/9 (44.4)	Reference		Reference	
**Severe**	61/127 (48.0)	2.282 (1.091–4771)	0.028*	2.319 (1.092–4.924)	0.029*	45/61 (73.7)	2.442 (0.882–6.765)	0.086		
**Critical**	142 /176 (80.7)	3.242 (1.588–6.620)	0.001*	3.537 (1.706–7.330)	0.001*	143/169 (84.6)	2.810 (1.011–6.921)	0.033*	2.120 (1.004–6.715)	0.025*
**Sepsis**										
**No**	143/262 (54.6)	Reference		Reference		130/170 (76.5)	Reference		Reference	
**Yes**	68/86 (79.1)	2.456 (1.675–3.602)	< 0.001*	1.354 (1.005–1.795)	0.043*	62/69 (89.9)	1.531 (1.160–2.764)	0.012*	1.414 (1.012–2.705)	0.032*
**Acute kidney injury**										
**No**	128/226 (56.6)	Reference				117/152 (77.0)	Reference			
**Yes**	83/122 (68.0)	1.367 (1.037–1.802)	0.027*			75/87 (86.2)	1.463 (0.952–2.247)	0.083		
**HAS-BLED score**										
**≥ 1 vs < 1**	190/307 (61.9) vs 21/41 (51.2)	1.267 (0.942–1.693)	0.119			171/212 (80.7) vs 21/27 (77.8)	1.203 (0.762–1.898)	0.427		
**≥ 2 vs < 2**	143/218 (65.9) vs 68/130 (52.3)	1.482 (1.110–1.980)	0.008*			125/153 (81.7) vs 67/86 (77.9)	1.250 (0.928–1.684)	0.141		
**≥ 3 vs < 3**	65/88 (73.9) vs 146/260 (56.2)	1.368 (1.032–1.952)	0.015*			59/70 (84.3) vs 133/169 (78.7)	1.128 (0.829–1.535)	0.443		

*AC* anticoagulation, *CCI* Charlson Comorbidity Index, *CI* confidence interval, *COPD* chronic obstructive pulmonary disease, *COVID-19* coronavirus disease 2019, *HAS-BLED* Hypertension, Abnormal renal/liver function, Stroke, Bleeding, Labile International Normalized Ratio (INR), Elderly, Drugs or alcohol use, *HR* hazard ratio

*Significant at *p* < 0.05

We then stratified all the patients into subgroups with various cutoffs of elevated D-dimer levels (500–7000 ng/mL). No significant difference of mortality was found between the two AC intensities in any subgroups of D-dimer scales (Table 3).

**Table 3:** The association between empiric therapeutic AC and mortality of patients with COVID-19 based on the D-dimer level

D-Dimer level (ng/mL)	N. of death/N. at risk (%)			Univariate analysis	
	Total	Therapeutic AC	Prophylactic AC	OR (95% CI)	***p*** value
**≥500**	150/230 (65.2)	57/92 (62.0)	93/138 (67.4)	0.788 (0.454–1.367)	0.397
**≥1000**	89/134 (66.4)	39/63 (61.9)	50/71 (70.4)	0.683 (0.332–1.402)	0.297
**≥2000**	65/103 (63.1)	29/49 (59.2)	36/54 (66.7)	0.725 (0.325–1.618)	0.432
**≥3000**	52/80 (65.0)	23/36 (63.9)	29/44 (65.9)	0.915 (0.364–2.302)	0.851
**≥4000**	37/59 (62.7)	16/29 (55.2)	21/30 (70.0)	0.527 (0.181–1.538)	0.239
**≥5000**	34/50 (68.0)	16/26 (61.5)	18/24 (75.0)	0.533 (0.158–1.799)	0.308
**≥6000**	30/44 (68.2)	13/21 (61.9)	17/23 (73.9)	0.574 (0.159–2.066)	0.393
**≥7000**	26/39 (66.7)	11/19 (57.9)	15/20 (75.0)	0.458 (0.117–1.789)	0.257

*OR* Odds ratio, *CI* confidence interval, *Significant at *p* < 0.05

Furthermore, patients who had a HAS-BLED score of ≥2, were associated with a significantly increased risk of mortality (HR 1.482, 95% CI 1.110–1.980, *p* = 0.008) (Table 2). Additional analysis showed that a high HAS-BLED score (≥3) was associated with a significantly likelihood of major bleeding (incidence of major bleeding: 11.4% vs 6.1%; Odds ratio: 1.883, CI: 1.114–3.729, *p* = 0.016) (Table 4).

**Table 4:** The association between HAS-BLED score and major bleeding

HAS-BLED score cutoff	Major bleeding/N. at risk (%)		Univariate analysis	
	≥ cutoff	<cutoff	OR (95%CI)	***p*** value
**1**	23/307 (7.5)	3/41 (7.3)	1.046 (0.624–1.714)	0.488
**2**	12/130 (9.2)	14/218 (6.4)	1.256 (0.832–2.114)	0.103
**3**	10/88 (11.4)	16/260 (6.1)	1.883 (1.114–3.729)	0.016*
**4**	6/30 (20.0)	20/318 (9.2)	2.112 (1.152–4.421)	0.001*

*OR* Odds ratio, *CI* confidence interval; *Significant at *p* < 0.05

## Discussion

Our analysis suggested that empiric use of therapeutic AC in patients requiring invasive mechanical ventilation significantly improved survival after adjustment of age, gender, race, comorbidities, COVID-19 disease severity, and major complications. However, our results did not demonstrate any survival benefit with therapeutic AC in non-critically ill patients. Lemos et al. reported similar results in a randomized clinical trial where the use of therapeutic intensity enoxaparin resulted in improved gas exchange over time, decreased D-dimer levels, and subsequently higher rates of successful liberation from mechanical ventilation after respiratory failure in severe COVID-19 patients [26]. The trial only included mechanically ventilated patients with D-dimer concentration greater than 1000 μg/L, no older than 85 years-old without severe organ dysfunction prior COVID-19 infection (ESRD, Child B or C cirrhoris, Heart failure class III or IV, etc.) or patients with contraindication for full AC [26] (Table S1). In contrast, the American Society of Hematology guidelines panel suggested use of prophylactic intensity of AC over intermediate or therapeutic intensity of AC in COVID-19 related critical illness without suspected or confirmed VTE [12], but these recommendations are based on limited evidence. Tremblay et al. compared patients on AC prior to COVID-19 infection with patients without AC or antiplalete therapy, and matched them by propensity score method. The factor of AC did not reduce the risk for all-cause of mortality, mechanical ventilation or hospital admission [27]. Still, despite widespread use of therapeutic AC, there is a current paucity of data to support empiric use in COVID-19 patients with high pro-thrombotic risk. Thus, our findings may contribute towards the ever-growing body of evidence regarding AC use in COVID-19 disease and may further contribute to therapeutic guidance.

The precise mechanistic advantage of AC remains unclear. AC was presumed to provide benefit by partially blocking thrombin formation and thus dampen the overwhelming inflammatory response following lung tissue damage in acute respiratory distress syndrome (ARDS) [28, 29]. However, post-mortem studies showed that thrombi in COVID-19 often developed as a consequence of direct vascular damage associated with viral infection and severe inflammation [17]. If so, the role of therapeutic AC in this scenario might not provide greater benefit than standard prophylactic AC, yet raises bleeding risk [18]. Yet, while the therapeutic mechanism remains unclear, our findings demonstrated that therapeutic AC provided mortality benefit to ICU patients with critical COVID-19 disease, but overall, we observed increased adverse bleeding events. Therefore, since critically ill COVID-19 patients admitted to the ICU carried higher thromboembolic risks (especially if intubated, sedated, paralyzed, and immobilized) then therapeutic AC may be reasonably considered. However, while escalating AC intensity may improve outcomes, its use should be balanced with bleeding risks on a case-to-case basis to mitigate the hemorrhagic events.

Since AC likely carries benefits, yet brings an elevated risk of adverse bleeding, D-dimer levels were initially used as a biomarker to guide appropriate use of AC therapy. Studies have demonstrated a correlation between varying levels of D-dimer (ranging from > 1000 ng/ml to > 2590 ng/ml) and PE or mortality in patients with COVID-19 [2, 10, 30]. Early data from Tang et al. suggested that patients with markedly elevated D-dimer levels (> 3000 ng/mL or > 6-fold of upper limit normal) would likely benefit from a higher dose of AC even with a low risk of thromboembolism [14, 15]. In contrast, recent evidence recommended against using D-dimer level cutoffs as the sole criteria to guide the intensity of AC [5, 12]. Our results further demonstrated that D-dimer levels did not correlate well with improved primary outcomes with empiric therapeutic versus prophylactic AC. It is noteworthy that D-dimer is a sensitive biomarker to rule out VTE, however, it has low specificity and may be elevated due to ongoing activation of the hemostatic system with inflammation, sepsis, liver disease, etc. (all of which can all be encountered in COVID-19). As such, elevated d-dimer alone is not sufficient to guide AC intensity, rather the whole clinical scenario should be considered.

Compared to other studies, our results showed a higher frequency of major bleeding among patients receiving therapeutic AC [18, 31]. It is likely related to the fact that only the highest doses were used in our study cohort, instead of the intermediate doses seen in other similar studies [18, 31]. Interestingly, HAS-BLED scores may offer a promising tool to assess bleeding risk. This risk-assessment tool was initially developed to evaluate bleeding risk in anti-coagulated patients affected by atrial fibrillation [32], and later was applied for patients with acute VTE [33–35], acute coronary syndrome (ACS) [36, 37], intracranial hemorrhage (ICH) [38, 39], postoperative AC after cardiac and vascular interventions [40, 41], etc. A score of 0 indicates low risk, 1–2 indicates moderate risk, and ≥ 3 indicates high risk. Our post-hoc analysis consistently demonstrated that a higher HAS-BLED score was associated with worse outcomes, with a score of ≥3 correlated with a higher bleeding risk and a score of ≥2 associated with a higher mortality. We suggested that use of the HAS-BLED rating system may help assess for risk stratification and guide AC dose selection.

Several limitations should be noted in the present study [10, 11]. First, by nature being a single-center retrospective review, this study is limited by sample size and generalizability. Also, the decision to start therapeutic AC was at the discretion of the treating physician, hence we believe there might have been selection bias, which created a smaller untreated group of critically ill patients. We were unable to match for all critically ill patients receiving AC, so there may have been residual confounding despite our use of propensity matching methods. Finally, due to COVID-19 related logistic constraints of treating COVID-19 during a time when the hospital was under surge capacity, diagnostic imaging for VTEs especially CT scans were restricted, even for critically ill and mechanically ventilated patients [14, 15].

## Conclusions

Our results demonstrated that the empiric use of therapeutic AC conferred survival benefit to critically ill patients requiring invasive mechanical ventilation, but had limited benefit to non-critically ill patients hospitalized for COVID-19. Given the higher risk of mortality among patients with a high HAS-BLED score, we believe that a balanced assessment of AC benefits and bleeding risks is important before considering escalation of AC intensity.

## Supplementary Information

**Additional file 1.**

## Data Availability

All data generated or analyzed during this study are included in the manuscript and the supplementary material.
