# Supplementary material for: Empiric use of anticoagulation in hospitalized patients with COVID-19: a propensity score-matched study of risks and benefits
Source: Biomark Res. 2021 May 1;9:29. doi: 10.1186/s40364-021-00283-y (PMC8087886; doi:10.1186/s40364-021-00283-y)
Supplement: Supplementary file 1 — Additional file 1. [file 40364_2021_283_MOESM1_ESM.docx]

**Figure S1: Distribution of the estimated propensity score for patients receiving therapeutic AC (treated) or not (control) prior and after the match: scatter diagram (A); histogram (B)**


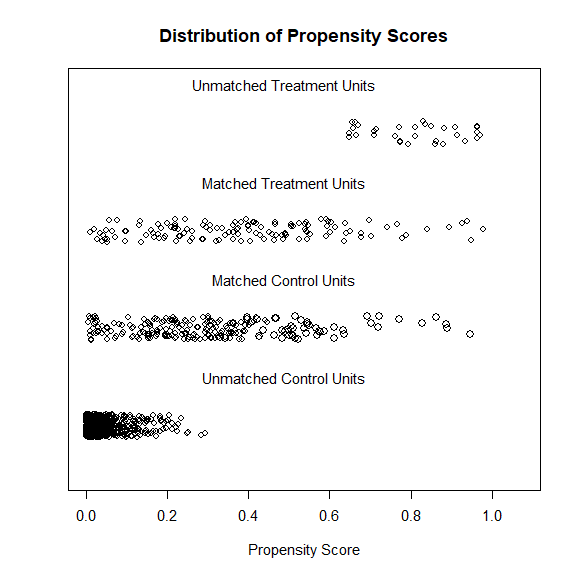


**A**

**
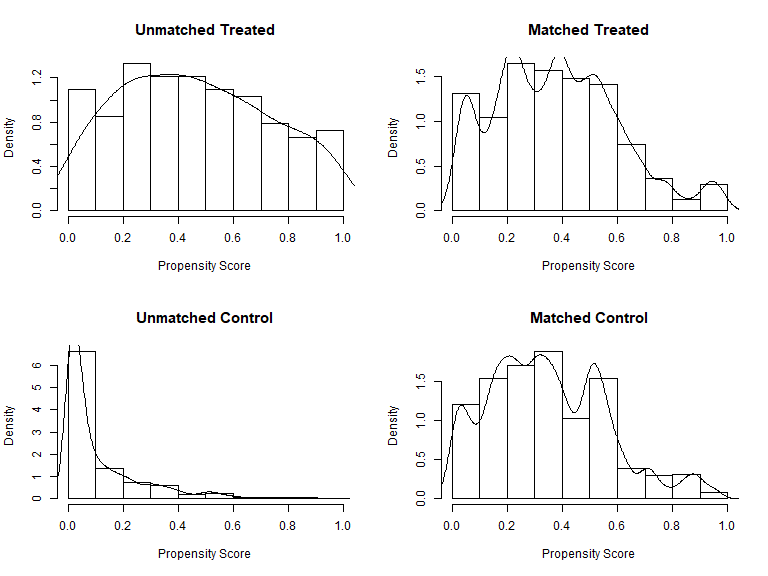
B**


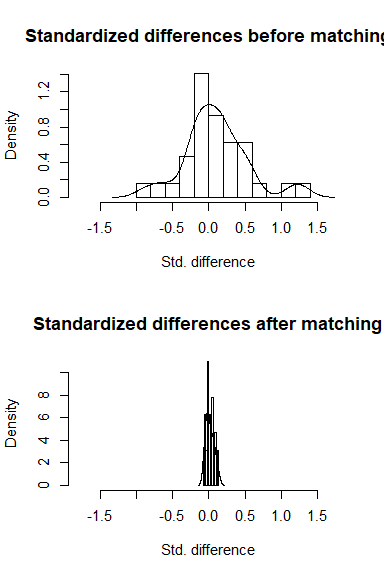
**Figure S2. Standardized mean differences in unmatched and matched sample.**

| Table S1: Comparison between HESACOVID trial and Lincoln study | | |
| --- | --- | --- |
|  | **HESACOVID study** | **Lincoln Medical Center Study** |
| Timeframe | April 2020 to July 2020 | March 5, 2020, to May 15, 2020 |
| Design | Randomized, controlled, open-label, phase II study | Retrospective case-control study  Analysis by subgroups |
| Matching technique | Randomization | Propensity score matching |
| Sample size | 20 patients | 973 patients |
| Inclusion criteria | - Greater than 18 years old - ARDS by Berlin definition due to COVID-19 infection - Under Mechanical ventilation - D-dimer > 1000 ug/L - INR < 1.5 - aPTT/ratio < 1.5 - Platelet >100 000/mm^3^ | - Greater than 18 years old - All Hospitalized for COVID-19 infection |
| Exclusion criteria | - >85 yo - Severe circulatory shock - Patients in RRT - Child B or C cirrhosis - Heart failure class III or IV - Advanced dementia - Disability from CVA - Recent stroke - COPD on O2 at baseline - Blood dyscrasias - Indication for anticoagulation given thrombotic event - Pregnancy | - Death or discharged within 48 hours after admission - Transferred from another facility - Long term anticoagulation before COVID-19 infection - Indication for anticoagulation given thrombotic event - Pregnancy |
| Intervention | Intervention 1: Therapeutic anticoagulation with Enoxaparin adjusted by renal function and age  Intervention 2: Prophylactic anticoagulation with Enoxaparin or Unfractionated heparin adjusted by renal function and age. | Group 1: therapeutic anticoagulation with Enoxaparin, apixaban, Unfractionated heparin, or fondaparinux adjusted by renal function and age.  Group 2: prophylactic anticoagulation with Enoxaparin. |
| Duration of the intervention | At least 96 hours no longer than 14 days | At the discretion of the treating physician |
| Outcomes | In the therapeutic anticoagulation group   - Improve PaO2/FiO2 ratio. after seven days - Higher rate of ventilator liberation - Higher rate of ventilator-free days - Lower D-dimer concentration overtime - No survival benefits at day 28 | In the therapeutic anticoagulation group   - No mortality benefit in the overall analysis. - Mortality benefit in the mechanically ventilated subgroup. - Higher risk of bleeding events for a patient with HAS-BLED >= 2 |
